# Neuroendocrine effects of a single bout of functional and core stabilization training in women with chronic nonspecific low back pain: A crossover study

**DOI:** 10.14814/phy2.15365

**Published:** 2022-09-06

**Authors:** Marta Silva Santos, Poliana de Jesus Santos, Alan Bruno Silva Vasconcelos, Ana Carolina Amado Gomes, Luciana Maria de Oliveira, Patrícia Rodrigues Marques Souza, Juan Ramón Heredia‐Elvar, Marzo Edir Da Silva‐Grigoletto

**Affiliations:** ^1^ Department of Physical Education, Functional Training Group Federal University of Sergipe São Cristóvão Brazil; ^2^ Department of Physical Education, Functional Training Group University Center Ages Paripiranga Brazil; ^3^ Institute of Biological Sciences, Laboratory of Immunology and Genomics of Parasites Federal University of Minas Gerais Belo Horizonte Brazil; ^4^ Department of Morphology, Laboratory of Entomology and Tropical Parasitology Federal University of Sergipe São Cristóvão Brazil; ^5^ Department of Health Education Federal University of Sergipe Lagarto Brazil; ^6^ Department of Physical Activity and Sports Science Alfonso X El Sabio University Madrid Spain

**Keywords:** activities of daily living, analgesia, exercise, opioid peptides, pain, spine

## Abstract

Exercise‐induced hypoalgesia (EIH) is characterized as the pain reduction after an exercise session and it seems to be related to the release of plasma β‐endorphin. In this sense, the core stabilization training (CT) has been suggested for patients with chronic nonspecific low back pain (CNSLBP), but it is unclear whether it induces EIH. Patients with CNSLBP have neuromotor dysfunctions that can affect the performance of functional tasks, thus, performing functional training (FT) could improve motor control and promote EIH, since functional training uses multi‐joint exercises that aim to improve the functionality of actions performed in daily life. EIH is usually assessed using quantitative sensory tests (QST) such as conditioned pain modulation, pressure pain threshold, and temporal summation. Thus, the sum of parameters from quantitative sensory tests and plasma β‐endorphin would make it possible to understand what the neuroendocrine effects of FT and CT session are. Our study compared the acute effect of CT and FT on the EIH and plasma β‐endorphin release, and correlated plasma β‐endorphin with quantitative sensory testing in patients with CNSLBP. Eighteen women performed two training sessions (CT and FT) with an interval of 48 h between sessions. EIH was assessed by QST and plasma β‐endorphin levels. Results showed that only FT significantly increased plasma β‐endorphin (FT *p* < 0.01; CT *p* = 0.45), which correlated with pain pressure threshold (PPT) and conditioned pain modulation (CPM). However, QST values were not different in women with CNSLBP after CT or FT protocols. Plasma β‐endorphin correlated with PPT and CPM, however, the same did not occur with a temporal summation.

## Graphical abstract

Women are more affected by chronic nonspecific low back pain; Women submitted to a single bout of functional training have increased plasma beta‐endorphin concentrations; Although it has increased plasma beta‐endorphin, a single bout of functional training did not cause exercise‐induced hypoalgesia.

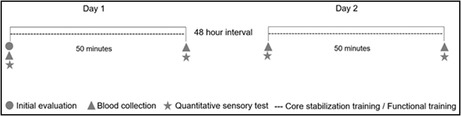

## 1 INTRODUCTION

Chronic low back pain is a common and prevalent lifelong health problem (Manchikanti et al., 2009). It is considered the public health problem with the greatest economic and social importance in the world, with global prevalence of approximately 40%, being more frequent in women (Airaksinen et al., 2006; Andersson, 1998; Blyth et al., 2001; Depintor et al., 2016; Vos et al., 2012). Women have higher pain rates and a higher risk of developing chronic pain and this has been associated with the decrease in gonadal hormones such as estrogen and estradiol, which reduce the number of μ receptors involved in the analgesic mechanism (Corrêa et al., 2015). In post‐menopausal women, the interrupted gonadal function causes a decrease in circulating estrogen levels (Baker et al., 2017), therefore, this population is more exposed to the development of chronic pain. Specific pathological causes of low back pain such as structural deformities, fractures, osteoporosis, tumor, and infection are rare and represent only 15% of cases (Hartvigsen et al., 2018; Koes et al., 2006) which classifies about 90% of cases as chronic nonspecific low back pain (CNSLBP) (Airaksinen et al., 2006; Maher et al., 2017). The disturbance in neuromotor activity appears to be a contributing factor in the transformation of acute low back pain into CNSLBP (Holm et al., 2002). This is because acute pain can induce kinesiophobia (Applegate et al., 2019), which can lead to alterations in the magnitude of the trunk and pelvis muscles activation (Becker et al., 2018), thus contributing to the transition from acute to chronic low back pain (Airaksinen et al., 2006; Merkle et al., 2020).

In this regard, physical exercise focused on the central region of the body and pelvis has been proposed, with the aim of improving the neuromotor recruitment of these regions, lumbar stability, and pain reduction (Frizziero et al., 2021). Thus, stabilization/motor control training, also referred to in the literature as core training/trunk‐specific training aims at training specific trunk muscles in order to improve the control and coordination of the spine and pelvis (Byström et al., 2013; Owen et al., 2020; Wang et al., 2012). The core training (CT) is effective in reducing pain in people with chronic low back pain, while aerobic exercises and combined modalities, i.e. including multiple types of exercise such as aerobic, resistance, and stretching, are not effective (Owen et al., 2020). Despite the trunk‐specific training being indicated for CNLBP and having demonstrated proven efficiency (Wewege & Jones, 2021), when moving in their daily lives, subjects with CNLBP perform global movements, such as sitting and standing. These global actions are multi‐artic, and therefore involve muscular activation not only of the trunk and pelvis, but also of the muscles of the lower limbs.

Nevertheless, the CNSLBP also causes the loss of function and decreased trunk motor control during basic work activities, such as sitting and standing up (Becker et al., 2018; Shahtahmassebi et al., 2017). Therefore, the use of training focused on global, multi‐joint, and multiplanar exercise, how the functional training that involves the activation of the trunk along with the appendicular skeleton could bring benefits on pain reduction, since training with these characteristics impacts on the increase of trunk strength and endurance (Da Silva‐Grigoletto et al., 2019). That being said, mimicking these actions by means of a global training that includes multi‐joint exercises could cause pain reduction.

Studies have shown an attenuation of pain perception after a single bout of exercise, this phenomenon is termed exercise‐induced hypoalgesia (EIH) (Koltyn, 2000, 2002; Koltyn et al., 2013, 2014; Wewege & Jones, 2021). EIH is commonly measured through quantitative sensory tests (QST), such as pain pressure threshold (PPT), temporal summation (TS), and/or conditioned pain modulation (CPM) (Leite et al., 2018). The EIH mechanism involves the activation of the endogenous opioid system during exercise (Bruehl et al., 2012; Koltyn, 2000). Recently, it has been suggested that plasma β‐endorphin can be used as a biomarker of pain intensity in patients with CNSLBP, as well as be used to evaluate the effects of physical exercise practice (Choi & Lee, 2019). Furthermore, despite the importance of investigating the mechanisms involved in EIH, few studies have examined it in people with CNSLBP (Kuithan et al., 2019), although exercise is recommended as a key treatment for the management of CNSLBP in international guidelines (Qaseem et al., 2017).

Therefore, to the best of our knowledge, investigation of the effects of CT and FT on pain inhibition pathways in humans has not yet been done. Together, QST's and plasma β‐endorphin levels would make it possible to understand the neuroendocrine effects of a single CT and FT session in patients with CNSLBP. Furthermore, it is important to understand the possible relationship between EIH and plasma β‐endorphin after CT and FT sessions. Thus, our objectives were: to compare the acute effect of CT and FT on EIH and plasma β‐endorphin release in patients with CNSLBP; and to correlate plasma β‐endorphin with QST's in patients with CNSLBP.

## 2 METHODS

### 2.1 Study design

**FIGURE 1:**
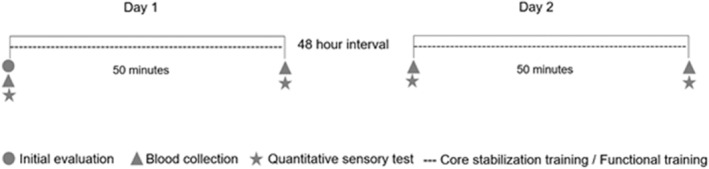
Study design

This was an evaluator‐blinded, randomized crossover study. Two types of intervention were performed, the CT and FT. Randomization was performed using a Latin square design, treatments were distributed so that each one appeared only once in each row. The subjects were randomly allocated into an intervention category (CT or FT), followed by 48 h of rest and then the performance of the opposite training protocol to that performed in the first moment. The evaluators of the quantitative tests and the analysis of plasma β‐endorphin were blinded to the type of intervention performed. The study design is shown in Figure 1.

The present research was carried out in the laboratory of the Department of Physical Education of the Federal University of Sergipe. The sample size calculation was performed using the G‐Power program (version 3.1.9.4), based on the results of two crossover model studies that measured plasma β‐endorphin and pressure pain threshold (Paungmali et al., 2017, 2018). It used 95% power and an alpha of 0.05, considering two conditions and two times. The result of the 16 measures, considering both groups.

### 2.2 Subjects

The study was conducted with women only, why they have higher pain rates and greater risk of developing chronic pain conditions. Exercise has been shown to be an effective treatment for this outcome (Greenspan et al., 2007). The clinical diagnosis of CNSLBP was issued by an orthopedic doctor and confirmed through anamnesis. The sample consisted of post‐menopausal patients between 45 and 59 years old, aiming to avoid interference from female sex hormones. To be included in the study, they needed to have had low back pain for more than 3 months, a pain level higher than three on the 11‐point numeric rating scale for pain (Corrêa et al., 2015, 2016; Hawker et al., 2011) body mass index (BMI) <30 kg/m^2^ and no history of spinal surgery. In addition, the volunteers could not practice physical exercise regularly, undergo physical therapy or other pain treatment, use analgesic medication, opioids or immunosuppressant, and anti‐inflammatory. Furthermore, we included patients considered sedentary or insufficiently active according to the International Physical Activity Questionnaire (IPAQ) (Lee et al., 2011).

Patients who missed the intervention at any time, who presented some psychiatric, motor or cognitive deficiency, auditory, visual or communication disorders that made it impossible to carry out the protocol were excluded. All volunteers were informed about the objectives and methods of the study, through oral and written exposure, and all of them signed an informed consent form. The study was approved by the local university committee (protocol no. 3.751.766).

### 2.3 Quantitative sensory testing

Four quantitative sensory tests were used to assess the pain process: Pressure pain threshold (PPT), temporal summation (TS), and conditioned pain modulation (CPM). In all tests, a digital pressure algometer with an area of 1 cm was used (EMG System).

The measurement of PPT was performed at two different sites, in the paravertebral and anterior tibial muscles. In the lumbar region (primary hypoalgesia), PPT was assessed bilaterally 5 cm from the lateral spinous processes of the third (L3) and fifth lumbar vertebrae (L5) (Corrêa et al., 2015). In the tibialis anterior muscle (secondary hypoalgesia) the measurement took place on the right leg at 5 cm from the tibial tuberosity (Corrêa et al., 2015). The pressure was increasingly applied and the patient was instructed to inform when the pressure clearly became painful. Three measurements were taken at each point, with a 30‐s interval between them and the arithmetic mean of the measurements used for statistical purposes. The PPT was evaluated by a physical therapist with 3 years of clinical experience in the care of patients with low back pain and a graduate degree in trauma‐orthopedics with an emphasis on manual therapy. All measurements were performed by the same investigator.

TS was evaluated with the algometer positioned on the volunteer's right arm at 7.5 cm above the wrist line, exerting a constant pressure of 4 kg/cm^2^. The volunteer was asked to verbally inform the pain intensity through the numeric rating scale for pain (Hawker et al., 2011), during the 1st, 10th, 20th, and 30th seconds of stimulation with the algometer pressing the point (Corrêa et al., 2015).

**FIGURE 2:**
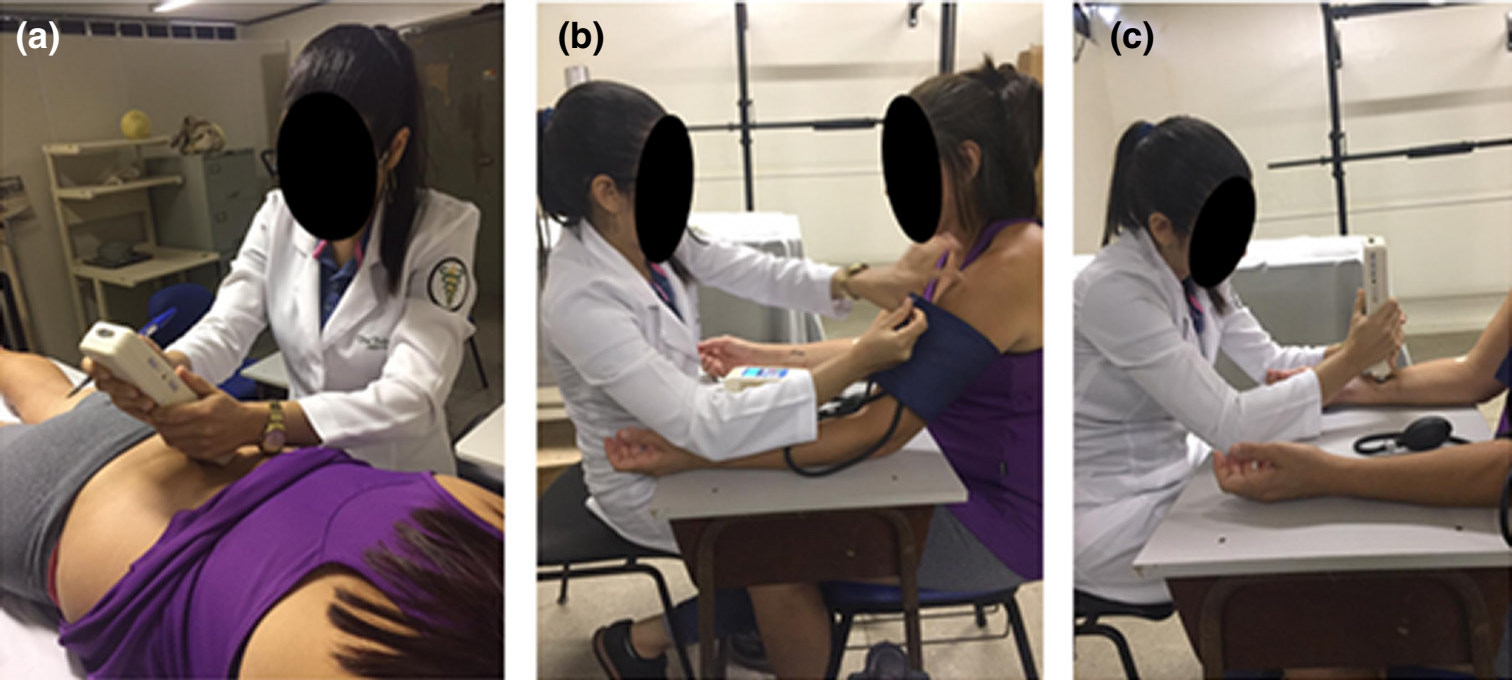
Evaluation of the quantitative sensory test. (a) Assessment of pressure pain threshold in the paravertebral musculature. (b) Application of the conditioned stimulus to assess the conditioned modulation of pain. (c) Evaluation of temporal summation.

To assess the CPM, firstly, the PPT was measured on the right forearm, 7.5 cm from the wrist line; then, ischemic compression of 270 mmHg was performed on the contralateral arm with a sphygmomanometer (Mikatos®), positioned 3 cm close to the cubital fossa (Figure 2). Pain intensity was verbally requested through the numeric rating scale for pain (Hawker et al., 2011) and when equal to or greater than 4, the PPT was measured on the right forearm at 7.5 cm from the wrist line, during ischemic compression. Five minutes after this procedure, the PPT was measured again, without compression this time (Corrêa et al., 2015).

### 2.4 Plasma β‐endorphin assessment

For the analysis of plasma β‐endorphin, a research diagnostic kit was used (Human β‐EP Beta‐Endorphin ELISA Kit) with the specificity of 9.38 pg/ml and detection rate of 15.63–1000 pg/ml. It was selected that the optical density (OD: wavelength of 450 ± 2 nm) measured by the spectrophotometer device was in picogram. A competition ELISA was used, in which the β‐endorphin present in the sample competes with an inhibitory antigen pre‐existing on the plate. The more β‐endorphin present in the sample, the less ODD is read by the spectrophotometer. Thus, the lower the picogram value, the more β‐endorphin is present in the sample. Following the manufacturer's instructions, arterial blood was collected and stored in an EDTA tube and the samples centrifuged for 15 min at 1000*g* at 2–8°C within up to 30 min after collection. The supernatant was collected and stored at −80°C. All samples were performed in duplicate and the mean used for statistical purposes.

### 2.5 Training protocols

Interventions were performed in a temperature‐controlled environment (23 ± 0.5°C), always in the morning, with 48 h between sessions, to minimize any residual effect (Paungmali et al., 2017, 2018). The protocols were applied by the same researcher, a physical education professional with a master's degree in physical education and 4 years of experience in applying the protocols. As a way to evaluate the perception of effort, we used the adapted BORG scale with a range of 0–10 (Dawes et al., 2005). This scale was applied at the beginning and at the end of both training protocols. Thus, both protocols were of moderate‐intensity (between 5 and 6 points).

#### 2.5.1 Core stabilization training

The CT protocol was based on the principles of stabilization, motor control, and trunk muscle strengthening (Boucher et al., 2016; Fulford et al., 2017). There was a warm‐up period, which lasted from 5 to 10 min, and consisted of performing hollowing and bracing maneuvers (Linde et al., 2017). Subsequently, the participants performed mobility exercises for the thoracic, lumbar, and hip regions, five sets of each.

The training protocol consisted of two moments: In the first, exercises were performed focusing on stability and motor control. For this, the bird‐dog plank exercises, side plank with support on both feet, bilateral hip thrust, side plank with support of one foot, static superman, and front plank were performed. In the second moment, the exercises aimed at training the muscles resistance, through the abdominal curl up and oblique exercises and hip flexion. Three sets of each exercise were performed and one exercise at a time. Muscle contraction time was 20 s with 40 s of rest. The entire training session lasted 50 min (Mueller & Niederer, 2020) and the sequence of specific trunk stabilization training exercises can be seen in Table 1.

**TABLE 1:** Description of the sequence of the core stabilization training exercises

Warm‐up period 5–10 min	Hollowing maneuver		5 reps
	Bracing maneuver		3 sets of 5 breaths
	Thoracic mobility		5 reps
	Lumbar mobility		
	Hip mobility		
Main part of training 35–40 min	First moment	Bird‐dog	3 series sustained for 20 s with 40 s rest
		Side plank with support on both knees	
		Bilateral hip thrusts	
		Side plank with one knee support	
	Second moment	Static Superman	
		Front plank	
		Curl up	3 series sustained for 20 s with 40 s rest
		Oblique	
		Hip flexion	

#### 2.5.2 Functional training

The same warm‐up period as for the CT was used for the FT protocol, in addition to the same time of execution and rest between sets. The protocol consisted in the performance of multi‐articular and dynamic exercises, which use large muscle groups such as the quadriceps and hamstrings for their execution (Bae et al., 2018). Exercises that mimicked activities such as sitting down and getting up from a chair, taking the stairs or pulling an object were chosen. In addition, we also used exercises that utilize the shoulder girdle (Tarnanen et al., 2012), and all the exercises used were aimed at using large muscle groups. Nevertheless, an alternation between exercises that used the upper and lower limbs was performed. During the entire exercise protocol, the patients were instructed to exhale in the concentric phase of the exercise and to inhale in the eccentric phase, maintaining an execution speed of about 3–4 s in each of the phases and respecting the difficulty of movement of each phase. In addition, during the performance of each exercise, the patients were instructed to maintain the normal curvatures of the spine. The detailed functional training session can be seen in Table 2.

**TABLE 2:** Description of the sequence of functional training exercises

Warm‐up period 5–10 min	Hollowing maneuver	5 reps
	Bracing maneuver	3 sets of 5 breaths
	Thoracic mobility	5 reps
	Lumbar mobility	
	Hip mobility	
Main part of training 35–40 min	Sit‐to‐stand exercise Bilateral resistance bands row Step‐ups (alternating the lower limbs) Vertical bench press with elastic bands Lunge Unilateral step‐up (without alternating the lower limbs) Open the elastic band (abduction with external rotation of the shoulder complex) Bilateral hip‐dominant squat Knee Push‐ups	3 series sustained for 20 s with 40 s rest

### 2.6 Statistical analysis

The data normality was verified through the *Kolmogorov Smirnov* test and the homogeneity of variances using Levene's test. For comparisons of variables between types of intervention (CT vs. FT) in relation to time (pre‐ and post‐intervention) a Repeated Measures ANOVA was used, followed by the Bonferroni post‐hoc. Pearson's correlation was used to relate the β‐endorphin variable with the PPT, TS, and CPM variables. For descriptive analysis, data were expressed as mean and standard deviation and data analysis was performed using SPSS® software version 22. The statistical significance level was set at *α* = 0.05.

## 3 RESULTS

Nine volunteers participated in the research and each participant performed the two training protocols (FT and CT), thus totaling 18 pre‐intervention and 18 post‐intervention measurements. The personal characteristics of the sample can be seen in Table 3.

**TABLE 3:** Main characteristics of the sample

Characteristics	Mean ± SD
Age (years)	52.72 ± 3.40
Weight (kg)	74.53 ± 15.31
Height (cm)	1.60 ± 0.06
BMI (kg/m^2^)	29.00 ± 5.30
Pain intensity (cm)	7.25 ± 3.24

**FIGURE 3:**
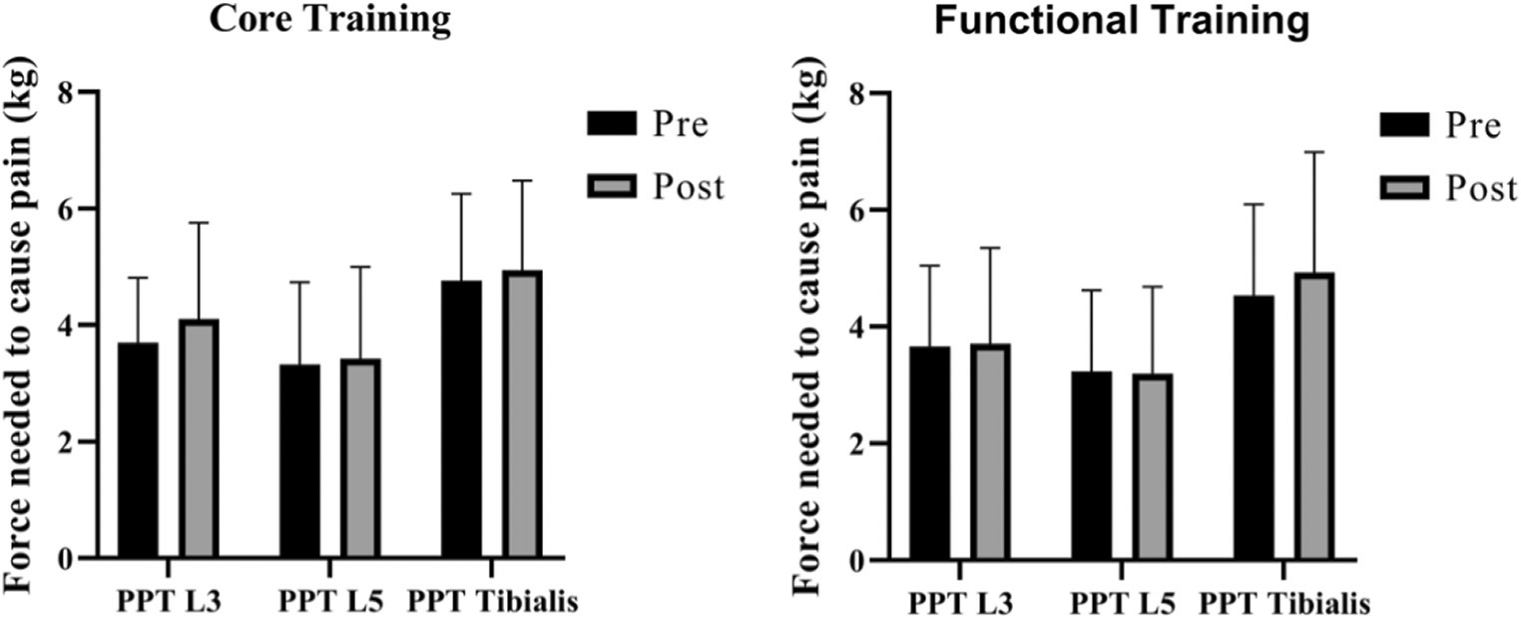
Comparison of the pressure pain threshold in patients with chronic nonspecific low back pain after performing a cross‐over design involving core stabilization training and functional training. The pressure pain threshold was measured at the level of L3, L4, and tibial anterior. L3, Lombar 3; L4, Lombar 4; PPT, pressure pain threshold; tibialis, Tibialis anterior.

**FIGURE 4:**
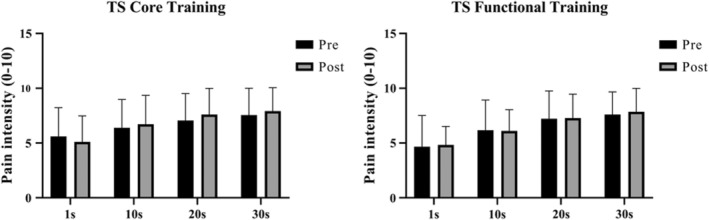
Comparison of temporal summation of pain in patients with chronic nonspecific low back pain after performing a cross‐over design involving core stabilization training and functional training. Pain intensity was evaluated at 1.10, 20 and 30 s after applying a constant pressure pain threshold.

**FIGURE 5:**
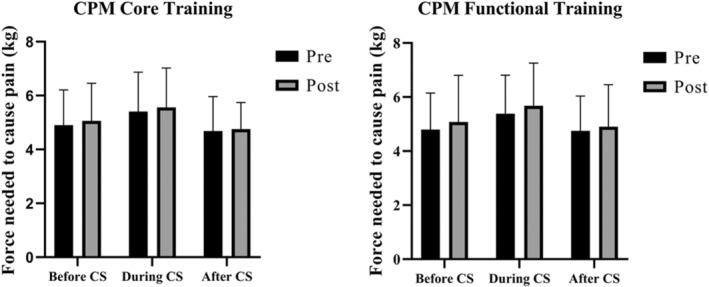
Comparison of conditioned pain modulation in patients with chronic nonspecific low back pain after performing a cross‐over design involving core stabilization training and functional training. The pressure pain threshold was measured before, during, and after the application of a conditioning stimulus. CPM, conditioned pain modulation; CS, conditioning stimulus.

Both training protocols did not significantly increase PPT of L3, L5, and tibialis anterior and no differences between groups were found (Figure 3). In the TS test, no significant intra‐ and inter‐group differences were observed in any of the times collected (Figure 4). Regarding CPM, there was no decrease in PPT during the application of the conditioning stimulus for both exercise conditions and times (pre and post intervention) (Figure 5). In addition, the CPM values before and after the application of the conditioning stimulus did not change. The sum of the outcomes of the PPT, ST, and CPM variables indicates that CT and TF groups did not induce EIH after a training session.

**FIGURE 6:**
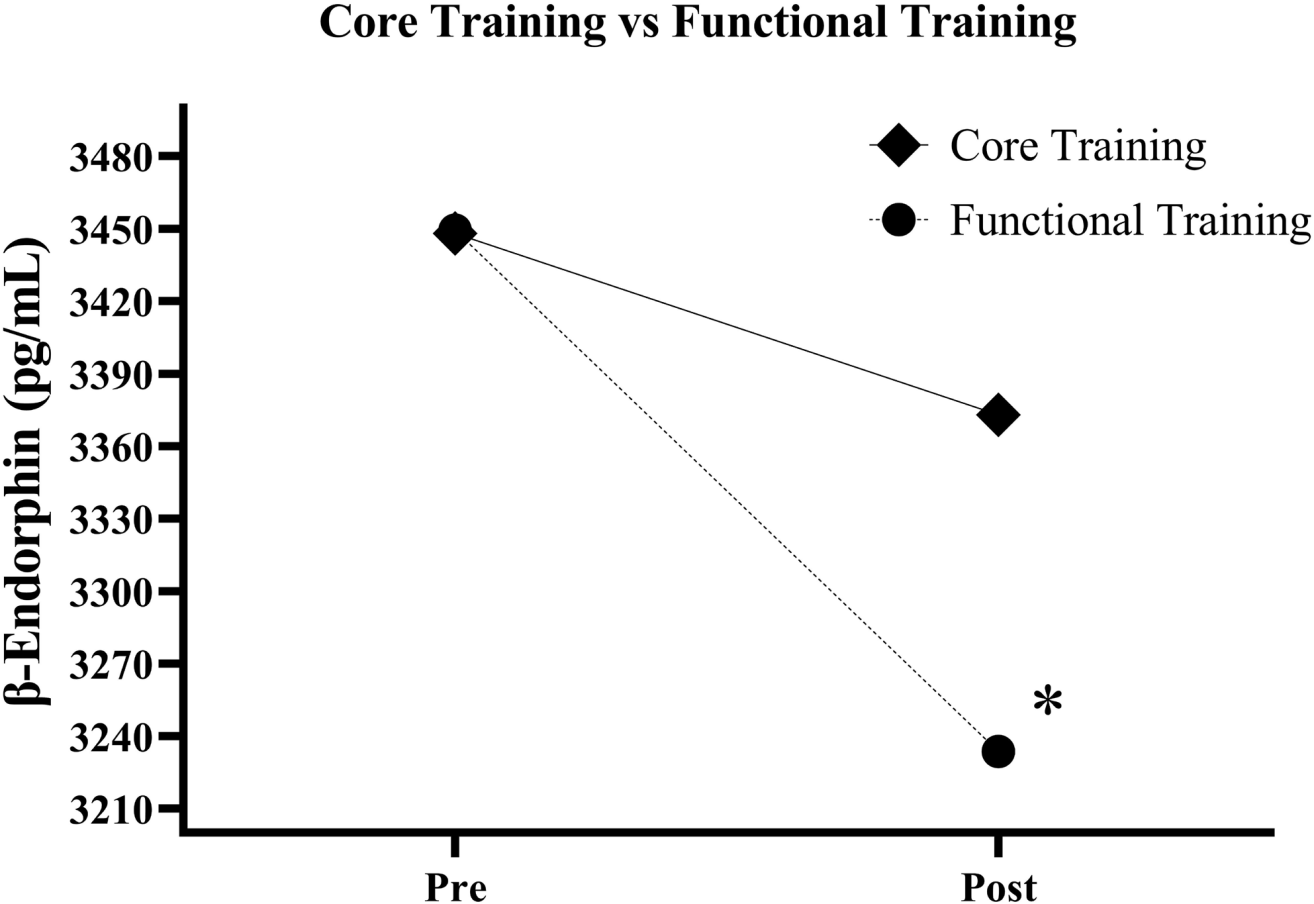
Comparison of plasma β‐endorphin concentration in patients with chronic nonspecific low back pain after performing a designer cross‐over design involving core stabilization training and functional training. The competitive ELISA technique indicates that the lower the value of β‐endorphin (pg), the higher the concentration of plasma β endorphin in the sample.

Plasma β‐endorphin increased significantly after TF application, the same did not occur for TC. There was no difference when the time × group factor was considered (Figure 6).

**FIGURE 7:**
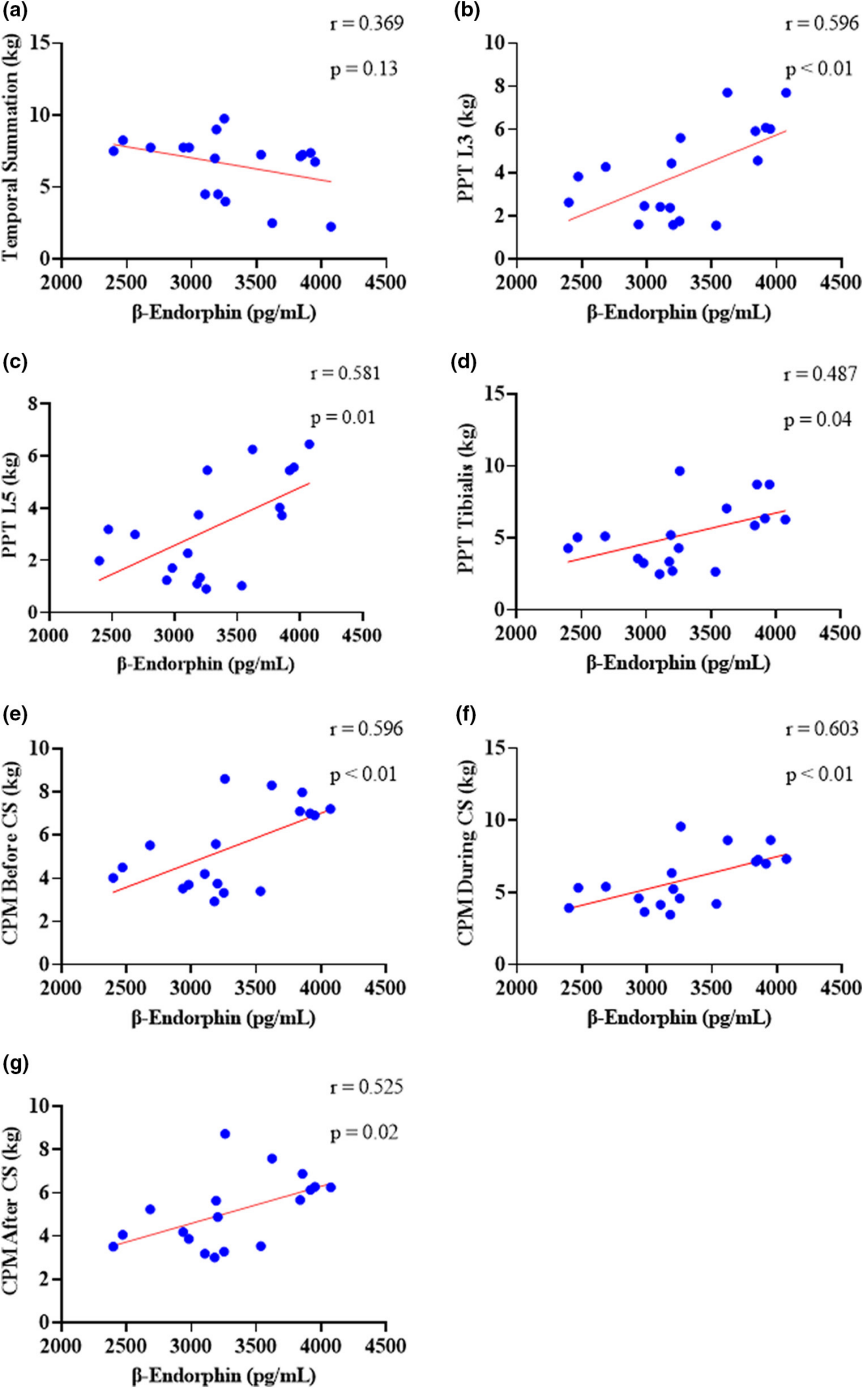
Correlation between plasma β‐endorphin and quantitative sensory tests in patients with chronic nonspecific low back pain. The competitive ELISA technique indicates that the lower the value of β endorphin (pg), the higher the concentration of plasma β endorphin in the sample. The graphs show a Pearson correlation between plasma β endorphin and: (a) Temporal summation; (b) PPT measured at L3; (c) PPT measured at L5; (d) PPT measured in tibialis anterior; (e) CPM before conditioning stimulus; (f) CPM during the conditioning stimulus; (g) CPM after conditioning stimulus. CPM, conditioned pain modulation; CS, conditional stimulus; PPT, pressure pain threshold; TS, temporal summation of pain.

Figure 7 shows the correlation between plasma β endorphin and each of the quantitative sensory pain tests: PPT, TS, and CPM. There was no correlation between β‐endorphin and TS. For all PPT points, which were measured at L3, L5, and tibialis anterior, we found a moderate and significant correlation with β‐endorphin, where the higher the PPT value, the lower the plasma β endorphin release. Regarding CPM, our results indicate that there was a moderate and significant correlation, in which the higher the CPM value during the use of the conditioning stimulus, the lower the plasma β endorphin release.

## 4 DISCUSSION

The main findings of this study showed that a FT session was able to increase the plasma β‐endorphin concentration in women with CNSLBP. However, CT and FT did not produce the phenomenon of EIH after a single training session. Furthermore, our results indicated that temporal summation is not related to the increase in plasma β‐endorphin. However, the higher the PPT, the lower the plasma β‐endorphin levels. And the more functional the CPM, the greater the release of this peptide into the plasma. Although there is evidence of the effects of CT on the improvement of stability, strength, muscle thickness (Wewege & Jones, 2021), and motor response time of the trunk muscles (Earde et al., 2014), little is known about the physiological mechanisms of this training on the modulation of ascending and descending pain pathways, remaining a topic of scientific interest. Therefore, we used different ways to assess the possible EIH effect through the PPT, TS, and CPM variables. Thus, after a single training session, both intervention conditions (CT and FT) did not cause EIH. Despite this, an increase in plasma β endorphin was found only in the FT group.

A randomized crossover study (Paungmali et al., 2017) investigated the effect of a CT protocol in patients with CNSLBP. The authors found a 7.64% increase in the PPT of the CT when compared to placebo or passive control, but with no significant intragroup difference. These findings corroborate our results, since we found an 11% increase in PPT of the CT group, but without a significant difference. It is known that PPT assesses the nociceptive threshold of nociceptors located in free nerve endings of sensory neurons. These neurons are located in the posterior horn of the spinal cord and are responsible for receiving mechanical stimuli and identifying them as a noxious stimulus (Stein, 2016). These nerve cells are the first neurons in the ascending pain pathway. Nociceptive information ascends through the lateral spinothalamic tract and reaches the thalamus where finally the noxious stimulus is interpreted as painful (Tracey & Mantyh, 2007). It is known that patients with CNSLBP have low PPT when compared to asymptomatic patients (Corrêa et al., 2015), which is termed as peripheral hypersensitivity and is part of the pathophysiological mechanism of CNSLBP. Thus, we believe that the fact that they present peripheral hyperalgesia prevents the EIH phenomenon from occurring in patients with CNSLBP, since the neurons in the posterior horn of the spinal cord are sensitive to excitatory substances, such as glutamate and substance P (Brito et al., 2017; Lima et al., 2017; Sluka et al., 2018). In addition, in subjects without CNSLBP, the FT session was able to promote EIH, which reinforces our justification (Matos Andrade Mesquita et al., 2019).

In the present study, we used two complementary quantitative sensory tests to the PPT, CPM, and TS. TS is the result of all responses from neurons located in the posterior dorsal horn of spinal cord (C‐nerve fiber) (Koltyn et al., 2013), which initiate ascending pain facilitation pathways. However, unlike PPT, this parameter involves inputting repetitive noxious stimuli at a constant intensity and measuring the level of pain facilitation through these stimuli. Improvement, that is, decrease in TS, is considered an important marker of central nervous system sensitization in patients with CNSLBP (Arribas‐Romano et al., 2020; Corrêa et al., 2015; Leite et al., 2018; Samuelly‐Leichtag et al., 2018). On the other hand, CPM is a psychophysical measure measured in humans and is correlated with the diffuse noxious inhibitory control (DNIC) mechanism (Lima et al., 2017), initially identified in rats and suggested as a phenomenon in which ‘pain inhibits pain’. (Kennedy et al., 2019). CPM assesses the ability of the nervous system to modulate a noxious stimulus, given the simultaneous application of a conditioning stimulus in a remote area. When a pain modulating system fulfills its physiological role of inhibition, the conditioning stimulus inhibits the pain felt during the test stimulus. In this way, TS and CPM are complementary, as they assess ascending and descending pain pathways, respectively.

Patients with CNSLBP usually have an increase in TS values and do not inhibit pain after application of a conditioning stimulus, which demonstrates a deficit in the CPM mechanism (Corrêa et al., 2015). Both situations can be observed in our sample and reflect a central hypersensitization, which is also part of the pathophysiology of CNSLBP. In this sense, a single session of CT or FT was not sufficient to change the ascending and descending pain pathways, since these pathways are not correctly working in patients with CNSLBP. Although the effect of EIH was not observed in a session of CT and FT, both protocols did not induce a worsening in somatosensory parameters, as found after exercise in other populations with chronic musculoskeletal pain (Rice et al., 2019). Thus, our training protocols are considered safe to be tested in randomized clinical trials, since they do not worsen peripheral and central hypersensitivity.

In addition to peripheral and central hypersensitivity, our patients were sedentary, a common aspect in the population of patients with CNSLBP. This could also justify the fact that both training did not promote EIH, since when exercise is regularly practiced, opioid receptors located in the periaqueductal gray (PAG) receive inhibitory stimuli from endogenous opioids, such as β endorphin (Lima et al., 2017; Sluka et al., 2018). These stimuli come from many different brain areas such as the anterior cingulate cortex, the insula, the hypothalamus, and the amygdala, and reaches the nuclei in the rostral ventromedial medulla (RVM), which in turn release serotonin in the posterior horn of the spinal cord, thus inhibiting the first‐order neuron (Brito et al., 2017). On the other hand, in sedentary people there is a lower endogenous opioid tone, that is, less endogenous opioids are released at the PAG area, while there is a greater density of serotonin receptors in the RVM, emerging on the cell surface and capture circulating serotonin, which results in a lower reception of serotonin by the first‐order neuron and consequently favors pain. In addition, in sedentary subjects, excitatory neurotransmitters, such as glutamate, cause pain facilitation (Brito et al., 2017; Lima et al., 2017; Merkle et al., 2020; Sluka et al., 2018). Thus, given that our sample consisted of women who were considered sedentary, who also did not have a functioning pain modulation system, the exercise protocols were not able to act at the level of the central nervous system, improving the opioidergic owner and regulating the CPM and TS. Nevertheless, it is important to emphasize that only a single bout of exercise was performed and that therefore this single dose was not able to provoke changes in the pain pathways and this does not preclude the protocols from being used in the long term to promote EIH.

Even without EIH outcome, only the FT group showed an increase in plasma β‐endorphin. It is known that physical exercise is able to stimulate the hypothalamus to release corticotropin‐releasing hormone (CRH). CRH acts on the anterior pituitary gland, which in response releases β‐endorphin into the bloodstream (Bruehl et al., 2017; Castro & Morrison, 1997; Guillemin et al., 1977; Solomon, 1999). FT protocol uses exercises that involve the activation of large muscle groups in the lower and upper limbs, which are activated concomitantly with the core muscles. Thus, by involving the use of a greater number of muscle groups, FT may have caused greater stress in the CNS, which responded with a greater release of β‐endorphin.

Furthermore, it's well established that neurons in the posterior horn of the spinal cord have opioid receptors, including the μ receptor (Machelska & Celik, 2020). β‐endorphin is an endogenous μ receptor agonist and acts by inhibiting first‐order neurons. Chronic low back pain is more common in women (Depintor et al., 2016; Vos et al., 2012) and this higher incidence is mainly associated with the decrease in gonadal hormones, such as estrogen and estradiol, which impact on the number of μ receptors that are involved in the analgesia process. Thus, since plasma β‐endorphin was elevated but the EIH did not occur, we hypothesized that μ receptors may be present in smaller amounts in the plasma membrane of first‐order neurons from patients with CNSLBP. Another explanation is that these receptors would be desensitized, therefore, a greater release of β‐endorphin would be necessary, which could be achieved with an increase in exercise intensity (Scheef et al., 2012).

In this perspective, we also verified the relationship between the release of plasma β‐endorphin and quantitative sensory tests. Thus, we found that plasma β‐endorphin release has no correlation with TS, but a moderate correlation between plasma β‐endorphin and PPT and CPM variables was observed. Thus, we believe that the higher the PPT values, the lower the plasma β‐endorphin concentration. We hypothesized that, in order for β‐endorphin to perform its inhibitory function, it must bind to the μ receptors present in the plasma membrane of the neuronal cell and consequently induce the EIH evaluated by the PPT. The lack of correlation between TS and β‐endorphin and the inverse relationship between CPM and β‐endorphin, suggests that the plasma release of this peptide during a single training session does not decrease central nervous system's excitability in patients with CNSLBP.

## 5 CONCLUSION

A FT session increased the plasma β‐endorphin concentration in women with chronic nonspecific low back pain, but this did not occur with a CT session. However, a single FT and CT session did not produce the phenomenon of exercise‐induced hypoalgesia. Plasma β‐endorphin is related to pressure pain threshold and conditioned pain modulation tests, but not to temporal summation.

## AUTHOR CONTRIBUTIONS

Marta Silva Santos: Conceptualization, Formal analysis, Methodology, Data curation, Writing. Poliana de Jesus Santos: Conceptualization, Methodology, Writing, Supervision. Alan Bruno Silva Vasconcelos: Formal analysis, Investigation, Writing. Ana Carolina Amado Gomes: Formal analysis, Visualization, Investigation. Luciana Maria de Oliveira: Formal analysis, Visualization, Investigation. Patrícia Rodrigues Marques Souza: Formal analysis, Visualization, Investigation, Methodology. Juan Ramón Heredia‐Elvar: Formal analysis, Visualization, Supervision. Marzo Edir Da Silva‐Grigoletto: Investigation, Methodology, Supervision, Data curation.

## FUNDING INFORMATION

This study was financed in part by the “Coordenação de Aperfeiçoamento de Pessoal de Nível Superior”—Brasil (CAPES)—Finance Code 001. Marta Silva Santos was a recipient of scholarship from CAPES.

## CONFLICT OF INTEREST

The authors have no competing interest to declare.

## ETHICAL STATEMENT

All participants were informed about ethical standards, objectives, procedures and risks related to the study and, after acceptance, signed the free, and informed consent form. The data were kept anonymous and the Committee of the Federal University of Sergipe approved the accomplishment of the present study (report no.: 3.751.766) and followed all the ethical aspects of the Declaration of Helsinki.
